# Learning by doing: To explore the influence of Simulation on Clinical Decision-Making Approaches on Final Year Medical Students at the University of Duisburg-Essen, Germany

**DOI:** 10.15694/mep.2021.000124.1

**Published:** 2021-05-13

**Authors:** Cynthia Szalai, Stephanie Herbstreit, Klara Novosadova, Susan Somerville

**Affiliations:** 1 Faculty of Medicine; 2 Faculty of Medicine; 3 Medical faculty; 4 Centre for Medical Education

**Keywords:** Clinical decision making, medical students, High-Fidelity Simulation, Learning Theories.

## Abstract

This article was migrated. The article was marked as recommended.

**Background:** Final year medical students at the University Duisburg-Essen, Germany, are unsatisfied with their clinical judgement skills in common elective and emergency clinical situations. A competency based medical curriculum determines that clinical judgement is an essential tool in effective patient care, patient safety and limiting clinical error. Approaches to clinical judgement include either analytical, intuitive or a combination of both approaches. Novices show specific factors, which are typical in inexperienced clinicians. Simulation provides opportunities in a competency-based medical education curriculum. There is limited evidence showing that simulation can provide an effective environment for teaching and learning clinical decision-making skills. This project explores how final year medical students at the University of Duisburg-Essen approach the clinical decision-making process as well as how simulation influences this process.

**Methods:** Ethics approval was obtained from the local ethics committee. After completing a 10-week simulation course,thirty-five students completed a clinical decision-making instrument to categorise their clinical decision-making approaches. The Novice Decision Making Model and the Cognitive Continuum Model were combined with learning theories in Simulation (Social Cognitive Theory) and used to explore and interpret data collected through questionnaires, interviews and observation.

**Results:** The majority (60%) of students employed a predominantly analytic approach, some students showed intuitive tendencies in clinical situations. During interviews students displayed typical novice approaches to decision-making and expressed positive comments relating to simulation.

**Conclusions:** Simulation presents an opportunity for teaching and learning clinical decision-making. Results show the need for further inquiry into learning clinical decision-making through simulation. This research provides initial evidence that simulation can be incorporated into curricular teaching of clinical decision-making.

## Introduction

Final year students in Germany are in the sixth year of their medical training, having completed their written exams but not their final oral exams ( MFT Medizinischer Fakultätentag der Bundesrepublik Deutschland e. V., 2015) and are only allowed to function in limited but supervised capacities on the ward. The German medical curriculum is still heavily seminar and lecture based ( Numbers, 1996; MFT Medizinischer Fakultätentag der Bundesrepublik Deutschland e. V., 2015; Zavlin *et al.,* 2017) offering limited opportunity for direct patient contact. Changes are being proposed to implement a competency-based curriculum based on the CanMEDS model of competency-based medical training but Personnel and financial constraints restrict the implementation of these changes in the training structure and do not take into account the time required for implementation of such a curriculum ( Nousiainen *et al.,* 2017). Students consistently report feeling inadequate in making clinical decisions and starting primary therapeutic measures. This feedback was not specific to students from the University of Essen. International students have reported similar dissatisfaction with teaching activities reported unpreparedness in undertaking the responsibility of decision-making, prioritisation and seeking help in clinical situations ( Manzar and Manzar, 2011; McGregor *et al.,* 2012).

As a teaching method simulation is especially relevant in today’s healthcare setting where there are ever-decreasing quality and frequency of patient contact for medical students due to factors such as time pressures, limited teaching situations and patient safety concerns ( Simpson and Courtney, 2002; Issenberg *et al.,* 2005; Peters and ten Cate, 2014). Evidence showed that high-quality simulation training might be equally effective as training in half of the traditional training hours ( Hayden *et al.,* 2014). Nishisaki ( Nishisaki, Keren and Nadkarni, 2007) reported a direct association between improved patient safety, simulation performance and management in clinical settings. Based on the supportive evidence in the literature for simulation as an effective teaching method with consequences for patient care, simulation is being increasingly integrated into the German medical curriculum ( Baschnegger *et al.,* 2017; Curry, 2018).

Increasing patient contact and subsequent curricular interventions has long been a goal of most universities ( Peters and ten Cate, 2014; Lin, Schillinger and Irby, 2015) with the expectation that conversion to a more practice-oriented curriculum would lead to an improved patient outcome. Teaching methods, which maximise learning outcomes but are also cost-effective are not only required but also expected by university management boards ( Maloney and Haines, 2016). Simulation may be a viable teaching and assessment method, offering a cost-effective option for implementation into the curriculum. van de Ven *et al.,* ( van de Ven *et al.,* 2017) showed that inter-professional medical simulation is a viable, cost-effective alternative where practice scenarios are used.

### Clinical Decision-Making (CDM)

Clinical decision-making can be defined as choosing between alternatives to provide the best possible patient care ( Banning, 2008). Clinical decision-making (CDM) is an essential skill for any competent doctor and has been shown to influence patient-outcome ( Nishisaki, Keren and Nadkarni, 2007; White, 2014). To facilitate the development of competent, autonomous healthcare providers, training programmes must include curricular aspects, which support the attainment of cognitive and intellectual skills required in decision-making ( Smith, Thurkettle and de la Cruz, 2004). CDM is a complex process which involves multiple components to understand how healthcare personnel undertake these daily decisions.

### Theories of Clinical Decision-Making

The hypothetico-deductive model is based on the recognition of cues from patient data, generation of a hypothesis followed by interpretation of these cues which leads to an evaluation of the generated hypothesis. Custers ( Custers, 2013)proposed that this model improves accuracy of clinical judgment in a controlled environment and enhanced rational, systematic problem solving. Another approach is intuitive-humanistic model defined intuition as knowledge without understanding ( Benner and Tanner, 1987). Rew, ( Rew, 2000) went into more detail to describe the model as the purposeful application of knowledge as a whole distinct from analytical reasoning process. Intuition is a component of complex judgment without the recognition of cues or conscious generation of hypotheses ( Benner, Tanner and Chesla, 1992; Lauri and Salanterà, 1995; Croskerry, 2015).

Combinations of these two models exist and include the Dual-processing theory proposed ( Djulbegovic *et al.,* 2012; Lambe *et al.,* 2016) as well as the Cognitive Continuum theory ( Hamm, 1988). Both theories propose that a practitioner does not engage with one approach to decision-making but can interchange between both approaches based on situational and inherent factors. O’Neil *et al.* ( O’Neill, Dluhy and Chin, 2005) also proposed the Clinical Decision-Making Model: (CDMM) which focusses on how novices engage in decision-making processes.

### Theories behind Simulation and Clinical Decision-making (CDM)

Simulation offers a valuable means to students when practising processes involved in decision-making in a safe, controlled environment ( Price *et al.,* 2017). Eva, ( Eva, 2005) proposes some of the challenges that teachers face when teaching clinical reasoning include, affording realistic scenarios, directed feedback and instruction to both analytical and intuitive processes as well as the possibility to observing expert behaviour. Simulation can provide all of the above as well as exploratory experiences which support experiential learning of clinical reasoning as well as the opportunity for all these factors to be developed as well as assessed ( Rutherford-Hemming, 2012; Zulkosky *et al.,* 2016; Price *et al.,* 2017).

O’Neill, Dluhy, & Chin, ( O’Neill, Dluhy and Chin, 2005) postulated a theory of clinical decision-making in novices, whereby anxiety due to a conscious deficit in working knowledge could lead to flawed cue recognition and cognitive processing. Simulation offers a medium, to not only broaden the learner’s exposure to various situations (increasing working knowledge) but students, through feedback could also learn cue recognition and hypothesis evaluation. Simulation is not only limited to analytic reasoning but can teach students to value and enhance their intuitive reasoning while limiting diagnostic error ( Rutherford-Hemming, 2012; Zulkosky *et al.,* 2016; Lavoie *et al.,* 2017). A practiced environment where structured clinical situations can be presented to learners to guide cognitive processing. During the simulation experience, students assume various roles as active participants as well as observers. Irrespective of their roles, through the process of metacognition ( Eva, 2005; O’Neill, Dluhy and Chin, 2005) learners can demonstrate aspects of the social-learning theory as evidenced by self-efficacy and agency by evaluating their performances against standards resulting in motivation and reflection to improve performance ( Bandura, 2001; Burke and Mancuso, 2012). New knowledge is acquired expanding their working knowledge into patterns and clusters ( Burke and Mancuso, 2012; Rutherford-Hemming, 2012). Bandura in his social cognitive learning theory ( Bandura, 2001) proposed that peer observation allows learners to use forethought to predict outcomes and compare their performance to others. During feedback, students discuss cue recognition leading to decision-making and influencing patient outcome ( Burke and Mancuso, 2012). Flavell, ( Flavell, 1979) proposed that metacognition or the conscious awareness of learning which occurs during simulation can lead to better clinical judgement and in turn improve performance.

### Description of the Simulation Course

In 2018 a ten-session simulation course was proposed for final year medical students as an introduction to common clinical emergencies, and ward situations. The course included multi-disciplinary simulation scenarios, extensive video-assisted feedback and opportunities to practice relevant practical skills. Scenarios simulated a range of daily clinical ward encounters as well as some common emergencies (See Table 1 and 2.) Scenarios were developed with intensive input from consultants as well as correlation with learning objectives stipulated from the German Medical Council for medical training. During the briefing students were informed on the patient setting and medical status. Students were expected to take a relevant history, perform a goal-oriented examination and devise a short-term management plan. We provided students with drug and patient charts on request and all monitors and medications were made available. Opportunities for communication with a senior doctor or diagnostic facilities were also available as well as all time-realistic results from diagnostics such as Ultrasound images and blood gas results. All cases were modelled on actual clinical scenarios which had occurred. A thorough discussion of clips from the recording was used to guide the feedback process.

**Table 1:** Format of Simulation course

Briefing (10 mins)	
Simulation In Small Groups (3-4 Students, 15-20 mins)	Videorecording
Video-Assisted Feedback (40-60 mins)	
Skills-Training (30-40 mins)	

**Table 2:** Schedule Skills Training and Simulation

Case	Simulator	SHK
Acute Abdomen (Herbstreit)	Simulated patient	1 SHK
Breaking Bad News	Training with colleagues	1 SHK
Epistaxis	Simulated patient + Task Trainer	2 SHK
Haemorrhagic Shock (Szalai)	METI (high fidelity mannequin)	1 SHK
Trauma (Herbstreit)	Simulated patient	2 SHK
Chest Pain (Szalai)	Simulated patient	1 SHK
Urology	Simulated patient	2 SHK
Paediatric Emergency	Simulated patient + Kinder-Meti (high fidelity mannequin)	2 SHKs
Shortness of Breath	Simulated patient	1 SHK
Cardiac Arrest (Szalai)	METI (high fidelity mannequin)	3 SHKs
The disoriented patient	Simulated patient	2 SHKs

SHKs: Students who play the role of inexperienced nurses.

Simulated Patient: actors who are trained for specific roles.

## Methods

An interpretive methodology was used to explore and relate the two processes of decision-making in a clinical setting and simulation ( Audi, 2015). Full ethical approval was obtained from the University of Duisburg-Essen ethics committee.

### Sample size, selection and recruitment

The Simulation and Skills Training Course is mandatory for all students who remain at the University Duisburg-Essen in their final year of medicine. Forty students (from a total of 120 graduates) participated in the course over ten weeks from November 2018-January 2019, representing on average about 33% of all final year students in the cohort. Thirty-five students participated in the study. Sampling was therefore purposive. All study participants completed a clinical decision-making instrument and were then invited to the focus group interviews. Ten (23%) students volunteered for the interviews.

Lauri & Salanterà’s, ( Lauri and Salanterà, 1995; Lauri *et al.,* 2001) clinical decision-making tool is a 24 item-scoring questionnaire which assesses nursing students’ approach to decision-making. The questionnaire categorises the approach to clinical decision making on the cognitive continuum spectrum of analytic to intuitive, with quasi-rationality lying in the middle. The lowest and highest scores generated from the questionnaire, denoted analytical and intuitive decision-making respectively.

### Interviews

Four semi-structured interviews with a total of eight participants were performed and recorded by the author (two students did not attend the interviews and rescheduling was not an option for the other eight students). Interviews were similar in format, allowing free speech with the use of cues from the interviewers to generate explanations. Students were allowed to elaborate on similar themes from previous interviews. All research data collected were stored and handled, anonymously. No identifying markers were present on transcribed interview folders. A thematic approach with constant comparison in data coding was employed ( Braun and Clarke, 2006; Clarke and Braun, 2013). Two authors participated in the manual coding process.

## Results/Analysis

### Questionnaire

The following results (See Table 3) were obtained from the Clinical Decision-Making questionnaire tool n= 35.

**Table 3:** Clinical Decision-making Questionnaire attributes and approaches

Attribute	Incidence %	Incidence %	Incidence %
Gender	Male (48.6%)	Female (51.4%)	
Previous Experience *	Yes (9) 25.7 %	No (25) 71.3%	No response (1) 3%
CDM Approach	Analytical (21) 60 %	Quasi-Rational (13) 37%	Intuitive (1) 3%

* Previous clinical experience was defined as previously or presently working as nurse or paramedic.

Eight out of the thirteen students who were analysed as having a quasi-rational approach to clinical decision-making reported previous experience in nursing, paramedic fields or worked as student assistants in clinical courses. Experience time ranged from 10 years (as intensive care nurse) to three months (as a paramedic). The results show that the majority of students (60%) take an analytical approach to clinical decision-making.

### Interviews

Based on a thematic analysis of data generated from interviews, five general themes were generated. From these central themes, subthemes emerged, which illustrated and supported the main topics. As can be seen, some sub-themes were appropriate for more than one central theme. See Table 4 below.

For actual interview dialog please see the supplementary files for Supplementary Material 1.

**Table 4:** Themes and sub-themes

Themes	Sub-Themes
Personal Issues	Difficulty Focussing Anxiety Lack of Confidence Personal Reflection
Social factors	Response from colleagues and seniors Lack of experience with autonomy and responsibility
Approaches to CDM	Flexible approach to CDM Task Features Pre-encounter
Learning Strategies	Observer and participant exposure Reflection on Behaviour
Simulation Issues	Reflection on Behaviour Feedback from experts/colleagues (Response from colleagues and seniors) Ability to observe peers

**Personal Issues:** These illustrated concepts or opinions which students considered within their control or intrinsic to their personalities but influenced their decision-making processes.

Difficulty focusing on multiple aspects of the situation was both mentioned and observed in the simulation. Students reported that especially in high stress, emergency, novel or complex situations that they were unsure of which decisions should be made.

Anxiety: This subtheme was illustrated multiple times during interviews. Students expressed anxiety at the consequences of their actions based on wrong decisions:

Lack of confidence: This sub-theme was where students were unsure of their primary diagnosis.

Reflection of Behaviours: Students have insight into their decision-making processes and actively endeavour to address their deficits.

**Social Factors:** These represent external factors, which influenced how students make clinical decisions. Students defined these factors as issues over which they either had no control such as reactions from colleagues, or issues which were influenced by their medical training such as lack of experience with assuming responsibility.

Response from colleagues and patients: Final year students reported being under pressure to make correct decisions especially in the presence of patients.

Lack of experience with autonomy and responsibility: Many students expressed annoyance that the final year was the only period where they were suddenly expected to reach autonomous decisions.

**Approaches to clinical decision-making:** Many students although expressing a systematic approach to decision making did express some tendencies towards intuition especially in decisions where they were familiar or considered themselves knowledgeable.

Thus, flexibility in decision-making approach was indicated. Though most students illustrated an analytical approach, some students demonstrated that in the familiar situations there were some decisions, which they made intuitively but other decisions required a logical approach.

Influence of Task Features: This theme highlights circumstances related to a specific situation, which students perceived as influencing their decision-making abilities. Multiple comments were made relating to the time required/afforded to reach a decision. (See Supplementary Material)

Pre-Encounter Data: as alluded to in the subtheme Flexibility in decision-making approach, students indicated that their approaches to clinical judgement were influenced by their access to pre-encounter data.

The above three themes and sub-themes detailed above, directly relate to students’ clinical decision-making experiences. Additional themes emerged from the data related to the influence of simulation on clinical decision-making. These themes seem to explain how students connected tenets of the simulation and applied them to their decision-making skills.

**Learning Strategies**: Specific learning strategies related to the social-cognitive learning theory were explored. Data showed repetition of two major themes.

Observation vs. direct participation in the simulation.

The students elaborated on the differences between actually participating in the simulation or being an observer. Many students highlighted that the learning effect varied depending on which role they assumed in the simulation. All students unanimously agreed that both roles afforded a learning effect but that the effect was different. As observers, students reported being more relaxed. Thus, having a greater opportunity to consider their observations, relate these observations to their possible hypotheses and then compare the two hypotheses.

Reflection of Behaviour: reflection plays an integral role in the cognitive aspect of the social cognitive learning theory. Students displayed a significant reflective component concerning decision-making during the simulation.

### Simulation Issues

Students explored what they considered to be the advantages and disadvantages of simulation with respect to clinical decision-making.

The ability to reflect on their behaviour: On asking students what aspects of the simulation they thought was beneficial.

Assuming responsibility for a situation: Students were asked how they felt about being responsible for their decision-making in the simulation.

Feedback from experts: On asking students what aspects of the simulation they thought was beneficial.

## Discussion

For orientation purposes a short description of medical training at the University of Duisburg-Essen will follow. Studying medicine requires 6 years and three months, all course participants were in their eleventh or twelfth semester of training. Based on the admissions system policies on average about 25 % of students have to wait for a university place. During this waiting period, which can last up to six years, these students usually take up training in either nursing or as paramedics and qualify as having previous experience. On average over fifty percent of medical students work part time in the above fields or as student assistants (SHKs) ( MFT Medizinischer Fakultätentag der Bundesrepublik Deutschland e. V., 2015; Bundesministerium für Bildung und Forschung, 2017).

How do final year medical students at the University of Duisburg-Essen approach the clinical decision-making process?

Based on the clinical decision-making questionnaire administered ( Lauri and Salanterà, 1995; Razieh, Somayeh and Fariba, 2018) the majority of final year students (60%) reported using an analytical approach when having to make decisions. These results have been substantiated by the literature, as students would be classified predominantly as novices. Novices due to their inexperience and limited working knowledge ( O’Neill, Dluhy and Chin, 2005; Banning, 2008) tend to rely on hypothesis generation and evaluation to reach a feasible conclusion.

However, the questionnaire results showed that a significant number of students (30%) were flexible in their decision-making approaches depending on the familiarity of the situation or various task features such as time frame within which to make a decision. It should be noted that the majority of students (eight of the nine students) who reported previous experience (89%) were categorised as quasi-rational and the only student categorised as intuitive also had more than five years previous experience as a paramedic. The results show that five of the students with no previous medical experience also reported a quasi-rational approach to decision making. Suggesting that students tended to switch between both approaches depending on the situational task features ( Hamm, 1988; Lauri *et al.,* 2001). Croskerry and Eva ( Eva, 2005; Croskerry, 2017) have both proposed flexibility in using both an analytic and intuitive approach in teaching approaches to decision-making. The authors support that students should be taught both approaches to decision-making and not predominantly a hypothesis testing approach.

Interviews and observations also highlight common factors of student behaviour and beliefs concerning the Novice Clinical Reasoning Model (NCRM). Factors such as anxiety, lack of confidence, difficulty focussing on multiples points, undertaking responsibility and limited exposure to specific clinical situations were all highlighted by the students as playing significant roles in clinical decision-making. Students also reiterated that such task features as time limitations influenced which approach, they adopted in an emergency or how much pre-encounter data they have access too. Environmental factors such as access to feedback from colleagues or patient affirmation influenced their approaches. Such factors have been described in the literature as influencing decision-making in novices ( O’Neill, Dluhy and Chin, 2005; Parker-Tomlin *et al.,* 2017, 2018).

Thus, the themes of personal issues, social factors and approaches to clinical decision-making substantiate previous literature that final year medical students at the University Duisburg-Essen have similar approaches and circumstances as other novices when having to make decisions.

Students elaborated on various aspects of how they perceived that simulation and decision-making were interrelated. In keeping with Bandura’s social cognitive learning theory ( Bandura, 1986, 1993) most students expressed positive opinions related to intentionality, self-reactivity and self-reflection during the simulation. They identified from their simulation experiences what their perceived goals were and created opportunities during feedback and following simulation scenarios to achieve these goals. Students were active in internalising these experiences and applying them to future ward situations showing elements of critical thinking and mindfulness ( Smyth and McCabe, 2017). The simulation also afforded a positive team environment to improve working knowledge, decrease anxiety and increase exposure to unfamiliar situations. These factors all play a role in the transition from novice to experienced decision-making skills ( O’Neill, Dluhy and Chin, 2005). The simulation presented opportunities to access their intuitive decision-making skills as well as their analytical competencies.

Students elaborated on various aspects of how they perceived that simulation and decision-making were interrelated. In keeping with Bandura’s social cognitive learning theory ( Bandura, 1986, 1993) most students expressed positive opinions related to intentionality, self-reactivity and self-reflection during the simulation. They identified from their simulation experiences what their perceived goals were and created opportunities during feedback and following simulation scenarios to achieve these goals. Students were active in internalising these experiences and applying them to future ward situations showing elements of critical thinking and mindfulness ( Smyth and McCabe, 2017). The simulation also afforded a positive team environment to improve working knowledge, decrease anxiety and increase exposure to unfamiliar situations. These factors all play a role in the transition from novice to experienced decision-making skills ( O’Neill, Dluhy and Chin, 2005). The simulation presented opportunities to access their intuitive decision-making skills as well as their analytical competencies.

However, simulation is not without its drawbacks. Although most responses were positive, some students did express the inauthenticity of simulation and reported the experience as artificial. Walsh, ( Walsh, 2013) confirmed this view and further expressed that success in simulation did not translate to successful management in a real ward situation. Counter arguments respond that the simulation process must be carefully thought out to represent and achieve the identified learning objectives ( Cook, 2013; Bashan, 2016).

Study Limitations

The issue of bias should be considered as the tutors were known to the students from having taught on the course. Thus a Halo effect is also possible ( Cohen, Manion and Morrision, 2018). The Hawthorne effect ( McCambridge, Witton and Elbourne, 2014) must also be considered as students were aware that they were recorded. Despite small sample size the study provides a basis for further discussion.

## Conclusion

Many final year students at the University Clinic of Duisburg-Essen felt unprepared to tackle everyday emergency/elective clinical situations due to lack of confidence in their clinical decision-making skills. The results substantiate that simulation encourages and promotes clinical decision-making competencies, which in turn may prepare new doctors for their clinical roles. Results show the need for further inquiry into learning clinical decision-making through simulation. If successfully implemented simulation can be incorporated into curricular teaching of clinical decision-making.

## Take Home Messages

• Simulation offers medical students the opportunity to learn effective clinical decision-making skills.

## Notes On Contributors

**Dr. Cynthia Szalai** is a consultant anaesthetiast and clinical lecturer at the University of Duisburg-Essen, Essen Germany. She was involved in the study design, execution of the study, data analysis and writing of the manuscript. ORCiD: https://orcid.org/0000-0001-8308-3814

**Dr. Stephanie Herbstreit** is a consultant orthopaedic surgeon and clinical lecturer at the University of Duisburg-Essen, Essen Germany. She was involved in the execution of the study and writing of the manuscript.

**Mrs Susan Sommerville** is active in medical education and serves as supervisior for this Master’s dissertation project. She assisted in the study design and analysis.

**Ms Klara Novosadova** is a student assistant/peer teacher in the department of Anasthetics, University of Duisburg-Essen. She assisted in the translation and analysis of student interviews.

## Appendices

List of abbreviations:

Canadian Medical Education Directives for Specialists: CanMEDS

Clinical Decision-Making: CDM

Novice Clinical Reasoning Model: NCRM
