# Supplementary material for: Learning by doing: To explore the influence of Simulation on Clinical Decision-Making Approaches on Final Year Medical Students at the University of Duisburg-Essen, Germany
Source: MedEdPublish (2016). 2021 May 13;10:124. [Version 1] doi: 10.15694/mep.2021.000124.1 (PMC10939537; doi:10.15694/mep.2021.000124.1)
Supplement: Peer review history

# Reviewer report: Reviewer response for version 1

**DOI:** 10.21956/mep.18971.r27171

**Published:** 2021-07-12

**Authors:** Mildred López

**Affiliations:** 1 Tecnologico de Monterrey, School of Medicine and Health Sciences

This review has been migrated. The reviewer awarded 5 stars out of 5 I think the article is an interesting manuscript worth reading by other medical educators. I see the value of the authors explaining the theory and the framework that supports the study; I think not many papers do that. I also found it interesting that the authors included the structure of the sessions and the topics, so other schools can replicate them. I would love to read more, as the other reviewer mentioned, about the qualitative analysis. I noticed that it was part of a thesis project, so I am sure it was a matter of summarizing all the work in here.

Reviewer Expertise:

NA

No decision status is available

# Reviewer report: Reviewer response for version 1

**DOI:** 10.21956/mep.18971.r27172

**Published:** 2021-05-23

**Authors:** Leila Niemi-Murola

**Affiliations:** 1 University of Helsinki

This review has been migrated. The reviewer awarded 3 stars out of 5 This is an interesting article focusing on the influence of simulation on clinical decision-making. This is a hot topic, and many medical students have difficulties in learning this essential skill. The authors give a thorough presentation of the theoretical background of their study, which the reader truly appreciates. Forty out of 120 students participated the study. The course was mandatory, but 40/120 students participated. Perhaps the course in organized three times per year? The authors used a validated tool designed to assess decision-making and they had semi-structured interviews for eight participants. The authors don’t report if they were individual of focus group interviews. The questionnaire revealed that most students had an analytical approach on decision-making and five themes emerged from the interviews. The results were very interesting, but the connection and the summary of the results received using the questionnaire and the interviews remains vague.These interesting results inspired many thoughts and hopefully the authors elaborate these themes in their further studies.

Reviewer Expertise:

NA

No decision status is available
